# Reproductive Lifespan and Motor Progression of Parkinson’s Disease

**DOI:** 10.3390/jcm11206163

**Published:** 2022-10-19

**Authors:** Ruwei Ou, Qianqian Wei, Yanbing Hou, Lingyu Zhang, Kuncheng Liu, Junyu Lin, Tianmi Yang, Jing Yang, Zheng Jiang, Wei Song, Bei Cao, Huifang Shang

**Affiliations:** Department of Neurology, Laboratory of Neurodegenerative Disorders, National Clinical Research Center for Geriatrics, West China Hospital, Sichuan University, Chengdu 610041, China

**Keywords:** Parkinson’s disease, sex difference, reproductive factors, estrogen, motor progression

## Abstract

Objectives: Estrogen not only plays a key role in the decreased risk of Parkinson’s disease (PD) but also influences its severity. We aimed to explore the effect of the reproductive lifespan on the motor progression of PD female patients in a large prospective cohort study. Methods: A competing risk analysis with a Fine and Gray model on 491 female and 609 male patients with PD was conducted. We regarded the chance of faster motor progression (as measured by the Unified Parkinson’s Disease Rating Scale (UPDRS) III increasing by ≥16 points during follow-up) and the chance of death as competing risks. The reproductive lifespan was regarded as the variable of interest, while faster motor progression was set as the primary outcome. Results: In the multivariable competing risk analysis, the male sex was not significantly associated with faster motor progression (subdistribution hazard ratio (SHR) 0.888, 95% CI 0.652–1.209, *p* = 0.450), while a shorter reproductive lifespan was associated with faster motor progression in women (SHR 0.964, 95% CI 0.936–0.994, *p* = 0.019). Sensitivity analysis indicated that a shorter reproductive lifespan was also significantly associated with faster motor progression in the 48 female patients who reported menopause after the onset of PD (SHR 0.156, 95% CI 0.045–0.542, *p* = 0.003). A linear mixed model also revealed the significant main effects of a short reproductive lifespan on the higher UPDRS III score in PD female patients at the last visit (*p* = 0.026). Conclusions: Our study indicates that a short reproductive lifespan contributes to faster motor progression in PD female patients, which has important implications for understanding the role of endogenous estrogen exposure in female PD and is beneficial to select appropriate patients in clinical trials.

## 1. Introduction

Parkinson’s disease (PD) is a chronic, progressive neurodegenerative disorder caused by the loss of nigrostriatal dopaminergic neurons. One prominent observation in PD is the remarkable sex difference in epidemiological and clinical features of the condition. The incidence and prevalence of PD in males exceed those of females, with a large meta-analysis reporting a 1.5–2-times higher risk for developing PD and a roughly 2-year earlier disease onset in men than women [1,2]. Estrogen, a kind of gonadal steroid hormone, is reported to be a potential contributor to such discrepancies because animal models and cell culture studies have shown that dopaminergic neurons and the density and sensitivity of dopamine receptors may benefit from estrogen [3,4,5]. Some epidemiological studies [6,7] have observed that reduced physiologic estrogen levels increased the risk for PD in women. Further evidence has indicated that postmenopausal estrogen use decreased the risk for PD [8,9]. Additionally, a clinical trial found that postmenopausal estrogen supplementation can improve the motor disability of PD female patients [5]. A double-blind, placebo-controlled, crossover study on estrogen replacement therapy also reported that estrogen was effective in improving levodopa-induced peak-dose dyskinesia without worsening motor disability in PD [10]. The above evidence raises the possibility that estrogen may play a protective role in PD.

The reproductive lifespan is the time period between menarche and menopause, which can reflect the period in which a woman benefits from estrogen exposure [11]. To date, some cross-sectional studies have explored the association between the reproductive lifespan and the clinical features of PD. An observational study [12] found that a shortened reproductive lifespan was associated with a younger age of onset and more severe disease disability in female PD patients. Another recent observational study [13] also found that female reproductive factors were positively associated with a delay of disease onset of up to 30 months. Therefore, we hypothesized that female reproductive factors may contribute to slowing the disease progression of PD. Clarification of the mechanisms involved in the effects of estrogens on PD, particularly the neuroprotective effects, is crucial. In the present prospective study, we aimed to explore the effect of the reproductive lifespan on motor progression in a prospective cohort of Chinese female patients with PD.

## 2. Patients and Methods

The protocol of the present study was approved by the Ethics Committee of the Sichuan University West China Hospital. All participants provided written informed consent.

### 2.1. Study Participants

A total of 1100 PD patients (491 women and 609 men) from the Department of Neurology, West China Hospital of Sichuan University, between April 2009 and April 2020, who met the following inclusion criteria, were recruited for the prospective study: (1) with a PD duration of ≤3 years; (2) at the Hoehn and Yahr (H&Y) stage < 3; (3) without motor complications, including dyskinesia and motor fluctuation; and (4) without dementia. PD was diagnosed according to the United Kingdom PD Society Brain Bank Clinical Diagnostic Criteria for PD [14]. The clinical diagnosis for PD was determined by the Movement Disorders Society version of the clinical diagnostic criteria for PD [15] before we performed the data analysis. All patients were subjected to brain MRI scans to exclude other neurological disorders. Patients with atypical and secondary Parkinsonism, those who had any unstable diseases, and those who declined to be visited were excluded from the study.

All participants were invited to finish a face-to-face follow-up visit after enrollment (from April 2010 to September 2021), with an interval of at least one year. During follow-up, 64 patients withdrew informed consent, contact was lost with 75, and 119 died. Among the 119 patients who died during follow-up, 12 patients had finished at least one face-to-face re-interview. Thus, a total of 856 patients (468 men and 388 women) provided data on clinical outcomes.

### 2.2. Determination of Female Reproductive Factors

Female reproductive factors were recorded using a structured interview at baseline. We recorded the data on age at menarche, age at final menstrual period, number of children, and number of pregnancies. The age of menopause was defined as the age at 12 months after the last menstrual cycle (natural menopause) or the age that both ovaries were surgically removed by bilateral oophorectomy for the treatment of cervical, endometrial, or ovarian cancer (surgical menopause). By subtracting the age at menopause from the age at menarche, we calculated the reproductive lifespan in years. Based on the quartiles (32 years and 37 years), female patients were divided into three groups: patients with a short reproductive lifespan (≤32 years), patients with a middle reproductive lifespan (>32 years but ≤37 years), and patients with a long reproductive lifespan (>37 years).

Among the 491 female patients, 441 patients reported menopause at enrollment, and 50 patients had not yet reached menopause. Among the 441 patients who reported menopause, 48 patients experienced menopause after the onset of PD.

### 2.3. Clinical Assessments

At baseline, a standardized assessment for all patients was completed by trained neurologists in our movement disorders center. Demographic and clinical data, including age, age of onset, disease duration, body mass index (BMI), and therapeutic schedule, were collected. The LEDD was calculated based on a previous systematic report [16]. Only drugs that had been stably used for at least 1 month were included to calculate the LEDD. Patients who were considered to have hypertension or diabetes mellitus were based on a self-reported doctor-diagnosed history of these conditions or were using disease-related medications. Smoking history was defined by >15 “pack/years”, quantifying the packs smoked per day multiplied by years. Drinking was defined as an average alcoholic drink (≥50 mL) at least once per week lasting more than half a year. The body mass index (BMI) was calculated as body weight (kg) divided by height squared (m^2^). Dysautonomia was defined as patients who had either constipation, urinary symptoms, sexual dysfunction, or orthostatic hypotension.

The motor severity of PD was evaluated using the Unified Parkinson’s Disease Rating Scale (UPDRS) part III [17] and Hoehn and Yahr (H&Y) stage [18], with higher scores indicating more severe motor disability. During follow-up, patients were guided to withhold their antiparkinsonian medications for at least 12 h before motor assessments. One hundred and eighty-nine (17.2%) patients at baseline and two hundred and eight (24.3%) patients at follow-up were not assessed at the off-medication state, and thus, we estimated an “off” score by adding the difference value of the study population’s mean “off” scores and mean “on” scores to the patient’s “on” scores, as previously reported [19].

The Montreal Cognitive Assessment (MoCA) [20] was used to evaluate cognition, with lower scores indicating poor cognition. The Hamilton Depression Rating Scale (HAMD) (24 items) [21] and Hamilton Anxiety Rating Scale (HAMA) [22] were used to evaluate depression and anxiety, respectively. The prevalence of rapid eye movement sleep behavior disorder (RBD) was calculated based on the percentage of patients who obtained a score of ≥5 in the RBD screening questionnaire (RBDSQ) [23].

### 2.4. Definition of Faster Motor Progression

An increase of 2.5–5.2 points in the UPDRS III is considered a clinically significant difference [24]. In the present study, we defined faster motor progression as a ≥16-point increase in the UPDRS III (mean of 4 points per year) based on the mean follow-up period of 4.0 ± 2.3 years. Time to such an event was defined as the interval in years from the time at baseline to the time at the ≥16-point increase in the UPDRS III that was first monitored.

### 2.5. Statistical Analyses

All analyses were performed using R version 4.0.2 for Windows. Categorical variables were presented as counts with percentages. Continuous variables were reported as the means with standard deviation.

The reproductive lifespan was regarded as the variable of interest. The primary outcome of the study was faster motor progression. We regarded the chance of occurrence of faster motor progression and the chance of death as competing risks. Therefore, we plotted the cumulative incidence functions (CIF) for female and male patients as well as female patients in different reproductive lifespan groups using death as a competing risk. Subdistribution hazard ratios (SHR), estimated with a Fine and Gray model, demonstrated associations with cumulative incidence accounting for competing risks, where a ratio of >1 indicates a positive effect. A further multivariable competing risk analysis was performed to identify risk factors for faster motor progression. Confidence intervals (CI) were reported as 95%, and the threshold for statistical significance was *p* < 0.05.

To explore the abrupt change in the estrogen effect (at the time of menopause) on motor progression, we also conducted a sensitivity analysis on the subgroup who experienced menopause after the onset of PD in women (*n* = 48). To measure the effect of the reproductive lifespan on the rate of motor progression without pre-assumption, we additionally conducted a linear mixed-effect (LME) model using the quantitative UPDRS III scores at the last visit as the outcome and the reproductive lifespan as an independent variable with the interaction of time.

### 2.6. Data Availability

The data are available upon request to the corresponding author.

## 3. Results

### 3.1. Baseline Data

The baseline and outcome characteristics between the male and female patients as well as among the different reproductive lifespan groups in women are listed in Table 1. A total of 1100 patients (609 men and 491 women) were included in the study. The mean overall age of the patients at enrollment was 61.5 ± 11.2 years, with a mean age of onset of 60.0 ± 11.2 years and a mean disease duration of 1.6 ± 0.8 years. At baseline, the mean UPDRS III score was 25.5 ± 11.6, and the mean LEDD was 197.7 ± 220.8 mg/day.

In the female population, the mean reproductive lifespan was 34.5 ± 4.7 years. Based on the quartile of the reproductive lifespan, 122 patients were classified into the short-reproductive-lifespan group, 230 were in the middle-reproductive-lifespan group, and 89 were in the long-reproductive-lifespan group.

### 3.2. Clinical Outcomes

Of the 1100 patients, 119 (10.8%) died. The mean survival time from the onset of symptoms to death of the 119 deceased patients was 5.6 ± 2.4 years. In the 856 patients who had data on clinical outcomes, 201 (18.3%) had an observed increase of ≥ 16 points in the UPDRS III after a mean of 4.0 ± 2.3 years of follow-up. The mean change scores in the UPDRS III from baseline to the follow-up visit were 8.1 ± 11.7 (Table 1).

In the female population (*n* = 491), 41 (10.0%) patients died. The mean survival time from the onset of symptoms to death of the 41 deceased female patients was 5.6 ± 2.3 years. In the 388 patients who had data on clinical outcomes, 139 (35.8%) had an observed increase of ≥16 points in the UPDRS III after a mean of 3.9 ± 2.2 years of follow-up. In the 388 patients, the mean change scores in the UPDRS III from baseline to follow-up were 8.7 ± 11.6 (Table 1).

### 3.3. Sex Differences in the Progression of PD

**Figure 1:**
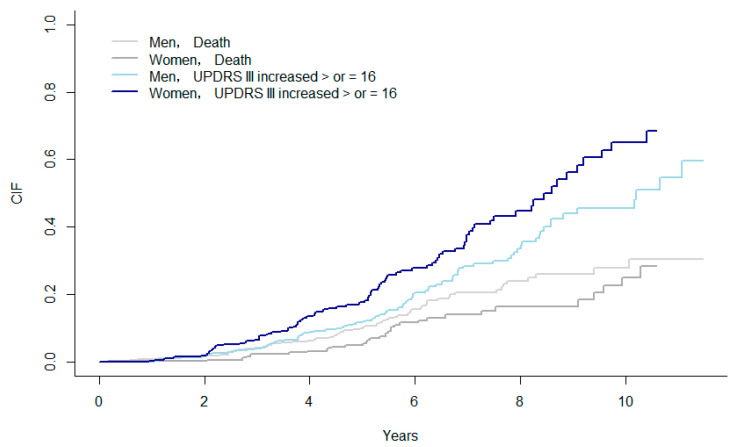
CIF between female and male PD patients. Note: CIF, cumulative incidence functions; UPDRS, Unified Parkinson’s Disease Rating Scale.

We regarded the chance of occurrence of faster motor progression and the chance of death as competing risks. Therefore, we plotted the CIF for the female and male patients and compared them using Fine and Gray. The CIF for a ≥ 16-point increase in the UPDRS III was significantly higher in the female patients than those in the male patients (*p* = 0.003) (Figure 1).

We regarded the chance of occurrence of faster motor progression (worsening UPDRS III) and the chance of death as competing risks. Therefore, we plotted the CIF for female and male patients and compared them using the Fine and Gray test. The median time to UPDRS III increasing by ≥16 points was significantly faster in the female patients than those in the male patients (*p* = 0.003).

After adjusting for age, age of onset, BMI, smoking history, diabetes history, hypertension history, constipation, RBD, and the UPDRS III score at baseline, the male sex was not significantly associated with faster motor progression (SHR 0.888, 95% CI 0.652–1.209, *p* = 0.450) in the multivariable competing risk analysis (Table 2).

### 3.4. Impact of Reproductive Lifespan on the Motor Progression of Female PD

**Figure 2:**
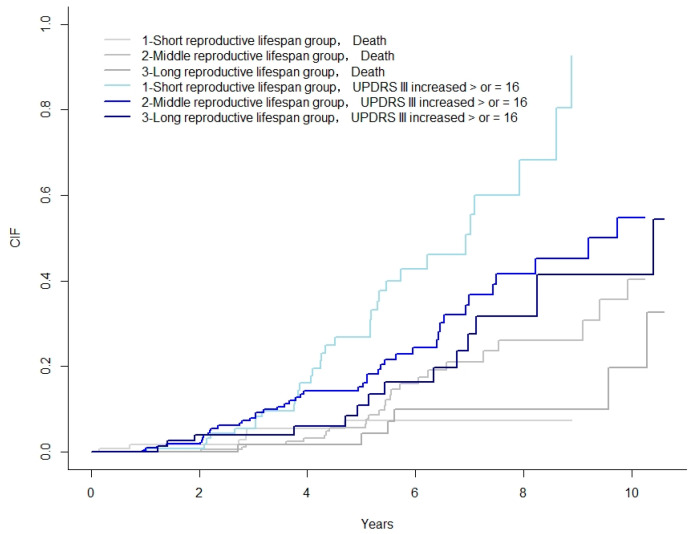
CIF for PD patients among different reproductive life span groups. Note: CIF, cumulative incidence functions; UPDRS, Unified Parkinson’s Disease Rating Scale.

We regarded the chance of occurrence of faster motor progression and the chance of death as competing risks. Therefore, we plotted the CIF for female patients with different reproductive lifespan groups and compared them using Fine and Gray. The CIF for a ≥16-point increase in the UPDRS III was significantly higher in the patients with a short reproductive lifespan than those in the patients with a long reproductive lifespan (*p* = 0.004) (Figure 2).

We regarded the chance of occurrence of faster motor progression (worsening UPDRS III) and the chance of death as competing risks. Therefore, we plotted the CIF for patients among different reproductive lifespan groups and compared them using the Fine and Gray test. The median time to a ≥16-point increase in the UPDRS III was significantly higher in the patients in the low-exposure group than those in the patients in the high-exposure group (*p* = 0.004).

After adjusting for age, age of onset, BMI, diabetes history, hypertension history, constipation, RBD, and the UPDRS III score at baseline, the reproductive lifespan in women was significantly associated with faster motor progression (SHR 0.964, 95% CI 0.936–0.994, *p* = 0.019) in the multivariable competing risk analysis (Table 2).

### 3.5. Sensitivity Analysis

The sensitivity analysis indicated that a short reproductive lifespan was also significantly associated with faster motor progression in the patients who reported menopause after the onset of PD (SHR 0.156, 95% CI 0.045–0.542, *p* = 0.003) (Table 2).

### 3.6. Linear Mixed Effect of Reproductive Lifespan

The results from the LME model show the main significant effects of a short reproductive lifespan on a higher UPDRS III score at the last visit (*p* = 0.026). Further, there were no significant group × time interaction effects in the model (*p* = 0.827) (Table 3).

In the linear mixed-effect model, the UPDRS III score at the last visit was set as the outcome, the reproductive lifespan as a fixed variable with the interaction of time, and age, age of onset, BMI, diabetes mellitus, hypertension, MoCA score, dysautonomia, RBD, and UPDRS III score at baseline as random variables.

## 4. Discussion

To the best of our knowledge, this is the first study to investigate the impact of the reproductive lifespan on motor progression in a large prospective cohort of PD female patients. One strength of our results is the large sample size, which is also likely to be representative of female patients with PD, with a wide range of age of onset and age. Another strength of our study is that we performed a competing risk analysis with sensitivity analysis to exclude the effect of death on clinical outcomes. To measure the effect of the reproductive lifespan on the rate of motor progression without pre-assumption, we also performed an LME model. We mainly found that short female reproductive lifespans were an independent risk factor for faster motor progression in PD female patients. Our study has important implications for understanding the role of endogenous estrogen exposure in the progression of female patients with PD. The findings of our study indicate it is necessary to select appropriate patients in clinical trials to explore the neuroprotective effect on PD.

The reproductive lifespan represents not only the years during which women can conceive but also the period in which women benefit from estrogen exposure [11]. The reproductive lifespan has been related to decreased morbidity, decreased mortality, and cardioprotection [25,26]. To date, the role of estrogen and its relevance to PD progression is not well-disclosed. According to previous observational studies [6,27], it can be speculated that exposure to estrogens may exert protective effects because related variables (longer estrogen stimulation, earlier age at menarche, and shorter deprivation due to pregnancies) have been associated with reduced risk of developing PD in women. In the present study, we observed that female patients with a shorter reproductive lifespan had faster motor progression as measured by the increase in the UPDRS III. Evidence of a possible role of estrogen replacement therapy on neurodegenerative disorders has already been recommended by the Women’s Health Initiative Memory Study, a randomized controlled trial where a twofold increased risk for probable dementia in postmenopausal women was observed among women who were exposed to estrogens plus progestin therapy [28]. To prolong stimulation, a randomized pilot trial found that postmenopausal hormonal therapy could improve the motor function of PD female patients [5]. In this context, our data support the observation result that endogenous estrogen exposure may be a protective factor for the dopamine system [12,29].

Gonadal steroid hormones have been identified to play a role in the nervous system [30]. Estrogen, in particular, has effects on the pituitary, hypothalamus, mesencephalic limbic system, nigro-striatum system, and other dopaminergic neurotransmitter system functions [31]. Furthermore, estrogen appears to protect against dopaminergic neuron loss in both disease and non-disease states [32,33]. It has been reported that estrogen can protect substantia nigra dopaminergic neurons in both male and female rodents against methyl-4-phenyl-1,2,3,6-tetrahydropyridine (MPTP)- and 6-hydroxydopamine (6-OHDA)-induced toxicity [34]. Estrogen also provides a protective effect by enhancing cell-mediated and humoral immunity [35,36] and has anti-inflammatory qualities in the setting of excessive inflammation [36]. Chronic neuroinflammatory processes are reported to play crucial roles in the neurodegeneration observed in PD [37]. Some studies have suggested that conditions resulting in reduced endogenous estrogen levels increase the risk, while treatments with estrogen decrease the risk of PD in women [6,7]. In addition, estradiol was demonstrated to exert antioxidant and neurotrophic properties, modulate neuronal plasticity, and decrease the degeneration of dopaminergic neurons [38]. Although exposure to endogenous estrogen may exert protective effects in PD patients, far less is understood regarding the effects of progesterone. Several experimental studies have suggested that the combination of estrogen plus progesterone appears to reverse the positive effects of estrogen alone [28].

It is reported that there is a stronger association between the UPDRS motor score and the measures of reproductive factors in female patients with early PD [12]. Thus, the relatively short observation period in our cohort cannot provide conclusions on the effect of the reproductive lifespan on long clinical outcomes. In addition, some patients (630/1100, 57.3%) received dopaminergic treatment at baseline. Therefore, it is reasonable to argue that potential interactions between hormones and pharmacological treatment may also affect disease progression. Further studies focusing on newly diagnosed, untreated patients with longer follow-up periods may help to clarify this issue.

In the present study, we did observe a sex difference in motor progression. However, a few studies in the literature have shown differences in the rate of motor progression, and most of them have suggested that estrogen has a protective effect [39]. One point worth discussing is that we did not report the number of children for male PD patients. Although having children does not change the male sex hormone levels, being fathers probably could also have an effect on subsequent motor progression in PD, such as more social engagement and having more purpose. Therefore, the lack of sex differences in motor progression in the current study could potentially be caused by hormone-related events in women with PD and by more social engagement and having more purpose as fathers in men with PD.

Some limitations should be discussed. Firstly, recall bias from information on women whose menarche and menopause occurred many years before data collection may exist in the present study. Secondly, the estrogen exposure time may not exactly reflect the “whole life” female endogenous estrogen because we did not record estrogen replacement therapy in the current study. Thirdly, we did not consider the effect of gynecological conditions, such as polycystic ovary syndrome and endometriosis, on the reproductive lifespan. Fourth, motor subtypes were not considered since not all patients’ motor scores were performed under the off-medication state.

## 5. Conclusions

Our study indicates that a prolonged reproductive lifespan may slow down the motor progression in female patients with early PD. Our study has important implications for understanding the role of endogenous estrogen exposure in the progression of PD female patients and is beneficial to select appropriate patients in clinical trials.

## Figures and Tables

**Table 1:** Baseline and outcome characteristics of the follow-up cohort.

Characteristic	Sex Group			Reproductive Lifespan Group			
	Total	Men	Women	Total	Short	Middle	Long
Number of samples	*n* = 1100	*n* = 609	*n* = 491	*n* = 441	*n* = 122	*n* = 230	*n* = 89
Number of children	-	-	1.8 ± 1.0	1.8 ± 1.0	1.9 ± 1.0	1.8 ± 1.1	1.6 ± 1.0
Age at menarche, years	-	-	14.5 ± 2.2	14.5 ± 2.2	16.0 ± 2.6	14.3 ± 1.8	13.1 ± 1.4
Age at menopause, years	-	-	49.0 ± 4.2	49.0 ± 4.2	44.7 ± 4.7	49.6 ± 1.8	53.2 ± 2.2
Number of pregnancies			3.0 ± 1.6	3.0 ± 1.6	3.2 ± 1.6	2.9 ± 1.4	2.7 ± 1.8
Reproductive lifespan, years	-	-	34.5 ± 4.7	34.5 ± 4.7	28.7 ± 4.3	35.4 ± 1.4	40.0 ± 2.0
Age at enrollment, years	61.5 ± 11.2	62.1 ± 11.7	60.9 ± 10.6	62.9 ± 9.1	63.1 ± 9.3	62.8 ± 9.6	62.7 ± 7.2
Age of onset, years	60.0 ± 11.2	60.5 ± 11.7	59.3 ± 10.6	61.3 ± 9.1	61.4 ± 9.2	61.3 ± 9.7	61.1 ± 7.2
PD duration at enrollment, years	1.6 ± 0.8	1.6 ± 0.8	1.6 ± 0.9	1.6 ± 0.9	1.6 ± 0.9	1.6 ± 0.9	1.6 ± 0.9
BMI at baseline, kg/m^2^	23.1 ± 2.9	23.4 ± 2.8	22.6 ± 3.1	22.6 ± 3.1	22.3 ± 3.1	22.4 ± 2.9	23.5 ± 3.3
Education, years	9.9 ± 4.2	10.5 ± 4.2	9.2 ± 4.1	9.3 ± 4.1	8.2 ± 4.3	9.6 ± 4.0	10.0 ± 4.2
Smoking	294 (26.7%)	290 (34.3%)	4 (0.01%)	2 (0.5%)	0	1 (0.4%)	1 (1.1%)
Dysautonomia	329 (29.9%)	191 (3.1%)	138 (28.3%)	134 (30.4%)	39 (32.0%)	75 (32.6%)	20 (22.5%)
RBD	340 (30.9%)	201 (33.0%)	139 (28.3%)	130 (29.5%)	35 (28.7%)	71 (30.9%)	24 (27.0%)
Hypertension	232 (21.1%)	137 (22.5%)	95 (19.3%)	94 (21.3%)	27 (22.1%)	42 (18.3%)	25 (28.1%)
Diabetes mellitus	84 (7.6%)	50 (8.2%)	34 (6.9%)	34 (7.7%)	12 (9.8%)	17 (7.4%0	5 (5.6%)
PD family history	117 (10.6%)	64 (10.5%)	53 (10.8%)	47 (10.7%)	13 (10.7%)	24 (10.4%)	10 (11.2%)
LEDD at baseline, mg/day	197.7 ± 220.8	199.3 ± 204.6	195.8 ± 239.9	199.7 ± 243.6	225.6 ± 327.1	189.1 ± 195.9	191.8 ± 220.3
MoCA at baseline	23.9 ± 4.3	24.3 ± 4.0	23.5 ± 4.5	23.4 ± 4.6	22.5 ± 4.9	23.6 ± 4.5	23.8 ± 4.1
HAMD at baseline	9.0 ± 7.8	8.1 ± 7.2	10.1 ± 8.4	9.9 ± 8.1	10.9 ± 8.2	9.8 ± 8.2	8.9 ± 7.7
HAMA at baseline	6.7 ± 6.2	5.9 ± 5.6	7.6 ± 6.7	7.7 ± 6.6	8.6 ± 6.4	7.7 ± 7.0	6.3 ± 5.8
Number of deaths during follow-up	119 (10.8%)	78 (12.8%)	41 (10.0%)	41 (9.3%)	7 (5.7%)	28 (12.2%)	6 (6.7%)
Survival time from symptoms onset, years	5.6 ± 2.4	5.6 ± 2.5	5.6 ± 2.3	5.6 ± 2.3	5.4 ± 2.3	5.6 ± 2.3	6.0 ± 2.5
UDPRS III at baseline	25.5 ± 11.6	27.1 ± 11.4	23.5 ± 11.5	23.7 ± 11.5	23.8 ± 11.3	23.9 ± 11.4	23.1 ± 12.1
Change in UPDRS III	8.1 ± 11.7	7.6 ± 11.9	8.7 ± 11.6	8.7 ± 11.5	9.8 ± 11.0	8.3 ± 11.9	8.1 ± 11.2
Faster motor progression	201 (18.3%)	100 (16.4%)	101 (20.6%)	90 (20.4%)	32 (26.2%)	44 (19.1%)	14 (15.7%)
Observation time for UPDRS III, years	4.0 ± 2.3	4.1 ± 2.4	3.9 ± 2.2	3.9 ± 2.2	3.6 ± 2.0	4.0 ± 2.1	4.2 ± 2.6
H&Y stage at baseline	1.9 ± 0.4	2.0 ± 0.4	1.9 ± 0.5	1.9 ± 0.5	1.9 ± 0.4	1.9 ± 0.5	1.8 ± 0.5

PD, Parkinson’s disease; BMI, body mass index; RBD, rapid eye movement sleep behavior disorder; LEDD, levodopa-equivalent daily dose; MoCA, Montreal Cognitive Assessment; HAMD, Hamilton Depression Rating Scale; HAMA, Hamilton Anxiety Rating Scale; UPDRS, Unified Parkinson’s Disease Rating Scale; H&Y stage, Hoehn and Yahr stage.

**Table 2:** Multivariable competing risk analysis for faster motor progression in PD.

Baseline Characteristics	UPDRS III Increase of ≥16 Points								
	Total Participants			Female Population			Patients Who Experienced Menopause after PD Onset		
	SHR	95% CI	*p*-Value	SHR	95% CI	*p*-Value	SHR	95% CI	*p*-Value
Female sex	0.888	0.652–1.209	0.450						
Reproductive lifespan				0.964	0.936–0.994	0.019 *	0.156	0.045–0.542	0.003 *
Age at enrollment	1.095	0.919–1.306	0.310	1.004	0.780–1.292	0.980	0.891	0.358–2.216	0.800
Age of onset	0.901	0.757–1.073	0.240	0.998	0.773–1.288	0.990	1.166	0.507–2.684	0.720
BMI	0.961	0.913–1.010	0.120	0.948	0.878–1.024	0.180	0.984	0.823–1.176	0.860
Smoking history	0.493	0.329–1.739	0.610						
Diabetes mellitus	1.132	0.659–1.945	0.650	1.007	0.415–2.442	0.990			
Hypertension	0.852	0.574–1.264	0.430	0.763	0.418–1.394	0.380			
MoCA	0.985	0.951–1.021	0.410	1.004	0.976–1.030	0.760	1.085	0.918–1.282	0.340
Dysautonomia	0.923	0.668–1.274	0.620	0.822	0.514–1.314	0.410	0.829	0.269–2.555	0.740
RBD	0.830	0.612–1.124	0.230	0.960	0.599–1.537	0.860	0.231	0.038–1.404	0.110
UPDRS III	0.960	0.947–0.973	<0.001 *	0.950	0.930–0.969	<0.001 *	0.927	0.859–1.001	0.052

PD, Parkinson’s disease; UPDRS, Unified Parkinson’s Disease Rating Scale; SHR, subdistribution hazard ratio; CI, confidence interval; BMI, body mass index; MoCA, Montreal Cognitive Assessment; RBD, rapid eye movement sleep behavior disorder. * Significant difference.

**Table 3:** Linear mixed effect of reproductive lifespan on the UPDRS III score at the last visit in the female PD population.

	UPDRS III Score at the Last Visit			
	Estimated Effect (β)	Standard Error (SE)	*t*	*p*-Value
Reproductive lifespan	−0.512	0.228	−2.242	0.026 *
Follow-up time in years	1.012	1.585	0.639	0.524
Reproductive lifespan × Follow-up time in years	0.010	0.045	0.219	0.827

PD, Parkinson’s disease; UPDRS, Unified Parkinson’s Disease Rating Scale; BMI, body mass index. MoCA, Montreal Cognitive Assessment; RBD, rapid eye movement sleep behavior disorder. * Significant difference.

## References

[1] Wooten G.F., Currie L.J., Bovbjerg V.E., Lee J.K., Patrie J. (2004). Are men at greater risk for Parkinson’s disease than women?. J. Neurol. Neurosurg. Psychiatry.

[2] Haaxma C.A., Bloem B.R., Borm G.F., Oyen W.J., Leenders K.L., Eshuis S., Booij J., Dluzen D.E., Horstink M.W. (2007). Gender differences in Parkinson’s disease. J. Neurol. Neurosurg. Psychiatry.

[3] Callier S., Le Saux M., Lhiaubet A.M., Di Paolo T., Rostene W., Pelaprat D. (2002). Evaluation of the protective effect of oestradiol against toxicity induced by 6-hydroxydopamine and 1-methyl-4-phenylpyridinium ion (Mpp+) towards dopaminergic mesencephalic neurones in primary culture. J. Neurochem..

[4] Shulman L.M. (2002). Is there a connection between estrogen and Parkinson’s disease?. Parkinsonism Relat. Disord..

[5] Gaikwad N.W., Murman D., Beseler C.L., Zahid M., Rogan E.G., Cavalieri E.L. (2011). Imbalanced estrogen metabolism in the brain: Possible relevance to the etiology of Parkinson’s disease. Biomark. Biochem. Indic. Expo. Response Susceptibility Chem..

[6] Ragonese P., D’Amelio M., Salemi G., Aridon P., Gammino M., Epifanio A., Morgante L., Savettieri G. (2004). Risk of Parkinson disease in women: Effect of reproductive characteristics. Neurology.

[7] Rocca W.A., Bower J.H., Maraganore D.M., Ahlskog J.E., Grossardt B.R., de Andrade M., Melton L.J. (2008). Increased risk of parkinsonism in women who underwent oophorectomy before menopause. Neurology.

[8] Currie L.J., Harrison M.B., Trugman J.M., Bennett J.P., Wooten G.F. (2004). Postmenopausal estrogen use affects risk for Parkinson disease. Arch. Neurol..

[9] Benedetti M.D., Maraganore D.M., Bower J.H., McDonnell S.K., Peterson B.J., Ahlskog J.E., Schaid D.J., Rocca W.A. (2001). Hysterectomy, menopause, and estrogen use preceding Parkinson’s disease: An exploratory case-control study. Mov. Disord..

[10] Nicoletti A., Arabia G., Pugliese P., Nicoletti G., Torchia G., Condino F., Morgante L., Quattrone A., Zappia M. (2007). Hormonal replacement therapy in women with Parkinson disease and levodopa-induced dyskinesia: A crossover trial. Clin. Neuropharmacol..

[11] Kang S., Park Y.M., Kwon D.J., Chung Y.J., Namkung J., Han K., Ko S.H. (2022). Reproductive Life Span and Severe Hypoglycemia Risk in Postmenopausal Women with Type 2 Diabetes Mellitus. Diabetes Metab. J..

[12] Cereda E., Barichella M., Cassani E., Caccialanza R., Pezzoli G. (2013). Reproductive factors and clinical features of Parkinson’s disease. Parkinsonism Relat. Disord..

[13] Frentzel D., Judanin G., Borozdina O., Klucken J., Winkler J., Schlachetzki J.C.M. (2017). Increase of Reproductive Life Span Delays Age of Onset of Parkinson’s Disease. Front. Neurol..

[14] Hughes A.J., Daniel S.E., Kilford L., Lees A.J. (1992). Accuracy of clinical diagnosis of idiopathic Parkinson’s disease: A clinico-pathological study of 100 cases. J. Neurol. Neurosurg. Psychiatry.

[15] Postuma R.B., Berg D., Stern M., Poewe W., Olanow C.W., Oertel W., Obeso J., Marek K., Litvan I., Lang A.E. (2015). MDS clinical diagnostic criteria for Parkinson’s disease. Mov. Disord. Off. J. Mov. Disord. Soc..

[16] Tomlinson C.L., Stowe R., Patel S., Rick C., Gray R., Clarke C.E. (2010). Systematic review of levodopa dose equivalency reporting in Parkinson’s disease. Mov. Disord..

[17] Goetz C.G., Fahn S., Martinez-Martin P., Poewe W., Sampaio C., Stebbins G.T., Stern M.B., Tilley B.C., Dodel R., Dubois B. (2007). Movement disorder society-sponsored revision of the unified Parkinson’s disease rating scale (MDS-UPDRS): Process, format, and clinimetric testing plan. Mov. Disord..

[18] Hoehn M.M., Yahr M.D. (1967). Parkinsonism: Onset, progression and mortality. Neurology.

[19] Ritz B., Rhodes S.L., Bordelon Y., Bronstein J. (2012). alpha-Synuclein genetic variants predict faster motor symptom progression in idiopathic Parkinson disease. PLoS ONE.

[20] Nasreddine Z.S., Phillips N.A., Bedirian V., Charbonneau S., Whitehead V., Collin I., Cummings J.L., Chertkow H. (2005). The montreal cognitive assessment, MoCA: A brief screening tool for mild cognitive impairment. J. Am. Geriatr. Soc..

[21] Hamilton M. (1967). Development of a rating scale for primary depressive illness. Br. J. Soc. Clin. Psychol..

[22] Clark D.B., Donovan J.E. (1994). Reliability and validity of the Hamilton Anxiety Rating Scale in an adolescent sample. J. Am. Acad. Child Adolesc. Psychiatry.

[23] Stiasny-Kolster K., Mayer G., Schafer S., Moller J.C., Heinzel-Gutenbrunner M., Oertel W.H. (2007). The REM sleep behavior disorder screening questionnaire—A new diagnostic instrument. Mov. Disord..

[24] Shulman L.M., Gruber-Baldini A.L., Anderson K.E., Fishman P.S., Reich S.G., Weiner W.J. (2010). The clinically important difference on the unified Parkinson’s disease rating scale. Arch. Neurol..

[25] Shadyab A.H., Macera C.A., Shaffer R.A., Jain S., Gallo L.C., Gass M.L., Waring M.E., Stefanick M.L., LaCroix A.Z. (2017). Ages at menarche and menopause and reproductive lifespan as predictors of exceptional longevity in women: The Women’s Health Initiative. Menopause.

[26] Brand J.S., van der Schouw Y.T., Onland-Moret N.C., Sharp S.J., Ong K.K., Khaw K.T., Ardanaz E., Amiano P., Boeing H., Chirlaque M.D. (2013). Age at menopause, reproductive life span, and type 2 diabetes risk: Results from the EPIC-InterAct study. Diabetes Care.

[27] Martignoni E., Nappi R.E., Citterio A., Calandrella D., Corengia E., Fignon A., Zangaglia R., Riboldazzi G., Pacchetti C., Nappi G. (2002). Parkinson’s disease and reproductive life events. Neurol. Sci. Off. J. Ital. Neurol. Soc. Ital. Soc. Clin. Neurophysiol..

[28] Shumaker S.A., Legault C., Rapp S.R., Thal L., Wallace R.B., Ockene J.K., Hendrix S.L., Jones B.N., Assaf A.R., Jackson R.D. (2003). Estrogen plus progestin and the incidence of dementia and mild cognitive impairment in postmenopausal women: The Women’s Health Initiative Memory Study: A randomized controlled trial. JAMA.

[29] Nitkowska M., Czyzyk M., Friedman A. (2014). Reproductive life characteristics in females affected with Parkinson’s disease and in healthy control subjects—A comparative study on Polish population. Neurol. Neurochir. Pol..

[30] Azcoitia I., Yague J.G., Garcia-Segura L.M. (2011). Estradiol synthesis within the human brain. Neuroscience.

[31] Cyr M., Calon F., Morissette M., Di Paolo T. (2002). Estrogenic modulation of brain activity: Implications for schizophrenia and Parkinson’s disease. J. Psychiatry Neurosci..

[32] Morissette M., Di Paolo T. (2009). Effect of estradiol on striatal dopamine activity of female hemiparkinsonian monkeys. J. Neurosci. Res..

[33] Liu B., Dluzen D.E. (2006). Effect of estrogen upon methamphetamine-induced neurotoxicity within the impaired nigrostriatal dopaminergic system. Synapse.

[34] Quesada A., Micevych P.E. (2004). Estrogen interacts with the IGF-1 system to protect nigrostriatal dopamine and maintain motoric behavior after 6-hydroxdopamine lesions. J. Neurosci. Res..

[35] Fischer J., Jung N., Robinson N., Lehmann C. (2015). Sex differences in immune responses to infectious diseases. Infection.

[36] Klein S.L., Flanagan K.L. (2016). Sex differences in immune responses. Nat. Rev. Immunol..

[37] Tansey M.G., Goldberg M.S. (2010). Neuroinflammation in Parkinson’s disease: Its role in neuronal death and implications for therapeutic intervention. Neurobiol. Dis..

[38] Saunders-Pullman R. (2003). Estrogens and Parkinson disease: Neuroprotective, symptomatic, neither, or both?. Endocrine.

[39] Reinoso G., Allen J.C., Au W.L., Seah S.H., Tay K.Y., Tan L.C. (2015). Clinical evolution of Parkinson’s disease and prognostic factors affecting motor progression: 9-year follow-up study. Eur. J. Neurol..

